# Predictive role of loneliness on mortality before the age 85 years among mid- to later-life adults in the United States: a 10-year retrospective cohort study

**DOI:** 10.1017/S2045796025100188

**Published:** 2025-09-11

**Authors:** Hui-Ying Fan, Mu-Rui Zheng, Qinge Zhang, Sha Sha, Yuan Feng, Zhaohui Su, Teris Cheung, Gabor S Ungvari, Lloyd Balbuena, Yu-Tao Xiang

**Affiliations:** 1 School of Nursing, Hainan Medical University, Haikou, HI, China; 2 Unit of Psychiatry, Department of Public Health and Medicinal Administration, Institute of Translational Medicine, Faculty of Health Sciences, University of Macau, Taipa, MO, China; 3 Centre for Cognitive and Brain Sciences, University of Macau, Taipa, MO, China; 4 Beijing Key Laboratory of Mental Disorders, National Clinical Research Center for Mental Disorders & National Center for Mental Disorders, Beijing Anding Hospital, Capital Medical University, Beijing, China; 5 School of Public Health, Southeast University, Nanjing, China; 6 School of Nursing, Hong Kong Polytechnic University, Kowloon, HK, China; 7 Section of Psychiatry, University of Notre Dame Australia, Fremantle, Australia; 8 Division of Psychiatry, School of Medicine, University of Western Australia, Perth, Australia; 9 Department of Psychiatry, University of Saskatchewan, Saskatoon, SK, Canada

**Keywords:** age factors, early mortality, loneliness, mid- to later-life population, oldest old

## Abstract

**Aims:**

Loneliness is a common public health concern, particularly among mid- to later-life adults. However, its impact on early mortality (deaths occurring before reaching the oldest old age of 85 years) remains underexplored. This study examined the predictive role of loneliness on early mortality across different age groups using data from the Health and Retirement Study (HRS).

**Methods:**

A retrospective cohort study was conducted using data from the 2010–2020 waves of the HRS, restricted to participants aged 50–84 years at baseline. Loneliness was measured using the 11-item UCLA Loneliness Scale, categorized into four levels: low/no loneliness (scores 11–13), mild loneliness (14–16), moderate loneliness (17–20) and severe loneliness (21–33). Cox proportional hazards models and time-varying Cox regression models with age as the time scale were created to evaluate the relationship between loneliness and early mortality, adjusting for sociodemographic, lifestyle, and physical and mental health factors.

**Results:**

Among 6,392 participants, the overall mortality rate before the age of 85 years was 19.1 per 1,000 person-years. A dose–response relationship was observed, with moderate and severe loneliness associated with 23% (adjusted hazard ratio [aHR]: 1.23, 95% confidence interval [CI] = 1.02–1.48) and 36% (aHR: 1.36, 95% CI = 1.13–1.65) higher mortality risk, respectively. Significant associations existed for the 65–74-year-old (aHR = 1.37, 95% CI = 1.03–1.83) and 75–84-year-old (aHR = 1.77, 95% CI = 1.23–2.56) age groups in the fully-adjusted models, but not for the 50–64-year-old age group. Time-varying Cox models showed a stronger association for severe loneliness (aHR = 1.65, 95% CI = 1.37–1.99).

**Conclusions:**

Loneliness is a significant predictor of mortality among older adults. Preventive and interventional programs targeting loneliness may promote healthy ageing.

## 1. Introduction

Loneliness refers to the distress experienced when there is a perceived discrepancy between an individual’s desired and actual social connections, often arising when existing relationships fail to meet expectations (Hawkley, 2022). Unlike social isolation, which refers to an objective lack of social contacts or networks, loneliness is a subjective experience. It is particularly common among mid- to later-life adults. According to the US National Poll on Healthy Aging, the proportion of adults aged 50–80 years who reported feeling socially isolated increased from 27% in 2018 to 56% during the COVID-19 pandemic in 2020. By early 2023, this figure remained notable, with over one-third of older adults in the United States reporting chronic loneliness (ongoing feelings of isolation and/or lack of companionship) (Malani *et al.*, 2023).

The Evolutionary Theory of Loneliness (Cacioppo and Cacioppo, 2018) proposed that when people feel lonely, they often feel less secure in their social and physical environment, which can make people more sensitive to possible social threats and lead them to expect negative interactions with others. Such expectations can influence social behaviours that reinforce feelings of isolation, which can trigger a range of negative psychological and biological responses. Studies also found that loneliness extends beyond emotional distress and has adverse health consequences. It interferes with mental health, leading to depression (Cacioppo *et al.*, 2010; Mann *et al.*, 2022), maladaptive coping behaviours, such as physical inactivity and alcohol abuse (Åkerlind and Hörnquist, 1992; Hawkley *et al.*, 2009), and accelerates cognitive impairment which is a major contributor to mortality among older adults (Huang *et al.*, 2024; Zhong *et al.*, 2017). Additionally, loneliness is linked to chronic physical illnesses such as diabetes, cancer, cardiovascular conditions and stroke (Office of the Surgeon General, 2023), which ranks among the leading causes of mortality in the U.S. population (Murphy *et al.*, 2021). Its health impact is comparable to smoking 15 cigarettes/day and exceeds the risks associated with obesity and physical inactivity (Holt-Lunstad *et al.*, 2017). Numerous studies, including a meta-analysis of 70 studies (Holt-Lunstad *et al.*, 2015), have shown an association of loneliness with an increased risk of all-cause mortality. Another meta-analysis of 90 cohort studies found that loneliness increased all-cause mortality by 14% and cancer mortality by 9% in the general population (Wang *et al.*, 2023).

Despite the abundance of evidence linking loneliness and overall mortality, its association with healthy aging is underexplored. Social and medical progress in recent decades have increased life expectancy at birth, and it was now approximately 73.5 years globally and 78.8 years in the United States in 2019 (Collaborators and Ärnlöv, 2020; GBD US Health Disparities Collaborators, 2022). Longevity is a fundamental human aspiration, and there is increased interest in the so-called Blue Zones of exceptional longevity (Kreouzi *et al.*, 2024). Longevity is often viewed as reaching the oldest old stage, typically defined as the age of 85 years or older, a widely accepted threshold for exceptional longevity in developed countries including the United States (National Research Council, 2001). Such longevity implies not only physical health but high levels of subjective well-being (Hitchcott *et al.*, 2018), so it is plausible that loneliness is a barrier to healthy ageing. Following prior research (Vaupel *et al.*, 2011), we defined early mortality as death before the age of 85 years in this study to focus on factors limiting survival to this milestone.

Developmental stages in life modify the relationship between loneliness and mortality. For instance, middle-aged adults (50–64 years) may experience loneliness differently from young-old (65–74 years) and old-old adults (75–84 years) due to differences in life circumstances, social roles and health conditions (Greer *et al.*, 2021). Middle-aged adults often face work-related stress, caregiving responsibilities or social transitions, which can exacerbate feelings of loneliness (Das and S Das, 2024 ). In contrast, older adults are more likely to experience widowhood, declining health and reduced mobility, all of which contribute to loneliness in distinct ways (Reynolds 3rd *et al.*, 2022). However, few studies have conducted stratified analyses to explore how loneliness affects mortality risk across different age groups (Surkalim *et al.*, 2022). Furthermore, many studies primarily focus on adults aged 65 years and above, overlooking middle-aged individuals.

To fill these gaps, this study aimed to (1) explore the association between loneliness and early mortality among mid- to later-life adults and (2) examine whether this association varies across different age groups.

## 2. Methods

### 2.1 Study design and samples

This was a retrospective cohort study based on the most recent 10-year dataset of the Health and Retirement Study (HRS), spanning from the 2010 to 2020 wave (Waves 10–15). The HRS, an ongoing nationally representative steady-state longitudinal study, has biennially surveyed U.S. adults aged 50 years and above since its inception in 1992 (Wave 1) (Sonnega *et al.*, 2014). The details of the methodology of the HRS have been previously described (Heeringa and Connor, 1995). This study made use of both HRS core data (including loneliness-related data) and exit data (including all-cause mortality data). Participants surveyed during the 2010 wave served as the baseline cohort and were followed electronically through the 2020 wave.

To be included in the study sample, participants were required to (1) be 50–84 years old and (2) have completed loneliness assessment at baseline. Individuals were excluded from our sample if (1) they had incomplete sociodemographic or health-related data that could not be imputed or (2) data were collected after their death date. Missing baseline data for sociodemographic, lifestyle or health variables were imputed using the last observation carried forward (LOCF) and next observation carried forward (NOCF) approaches as recommended (Lydersen, 2019; Nakai *et al.*, 2014). Ethical approval for the HRS was granted by the Institutional Review Board of Virginia Commonwealth University (No. HM20023839). Informed consent was obtained from all participants by the HRS investigators. As a secondary analysis of HRS data, the present study was exempt from ethical review.

### 2.2 Measurements

#### 2.2.1 Loneliness

Loneliness was measured using the 11-item UCLA (University of California, Los Angeles) Loneliness Scale (UCLA-11) (Lee and Cagle, 2017; Smith *et al.*, 2017), which is a short and validated version of the 20-item Scale (Version 3) (Russell, 1996). Participants rated their frequency of feelings including (1) lacking companionship, (2) left out, (3) isolated from others, (4) in tune with others, (5) alone, (6) having people they could talk to, (7) having people they could turn to, (8) understood by others, (9) close to others, (10) part of a friend group and (11) a sense of shared commonality with others around them. Responses were recorded on a 3-point Likert scale (1 = ‘often’ to 3 = ‘hardly ever or never’). Items 1, 2, 3 and 5 were reversely scored. Total scores ranged from 11 to 33, with higher scores indicating more severe loneliness. Following a previous study of cancer survivors (Zhao *et al.*, 2024), UCLA-11 total scores were classified into four levels based on quartiles of the distribution: 11–13 (low or no loneliness), 14–16 (mild loneliness), 17–20 (moderate loneliness) and 21–33 (severe loneliness), which enables the exploration of loneliness patterns and subgroup comparisons among mid- to later-life adults in the United States.

#### 2.2.2 Ascertainment of mortality before the age 85 years

Mortality data for each HRS wave were collected through exit interviews with next-of-kin and cross-referenced with the National Death Index, ensuring high identification rates (Weir, 2016). The outcome was defined as all-cause mortality occurring before the oldest old stage (≤85 years) (National Research Council, 2001), monitored from the 2010 wave to the 2020 wave. Given that HRS interviews in a given wave were not conducted within a single calendar year (e.g. the 2010 wave included interviews spanning 2010 and 2011), the follow-up period for each participant began in the month of their interview during the 2010 wave (conducted in 2010 or 2011) and ended in the month of their interview during the 2020 wave (conducted in 2020 or 2021) (Kezios *et al.*, 2023). Participants who died ≤85 years old had follow-up terminated at the date of death, while those who survived or died >85 years were censored upon reaching 85 years. Individuals who were alive and under the age of 85 years throughout the follow-up period were censored at the date of their 2020 wave interview, whereas those lost to follow-up under the age of 85 years were censored at the time of their final interview. Person-years were calculated from the start of follow-up to either death or censorship.

#### 2.2.3 Covariates

Sociodemographic and health-related variables potentially related to loneliness symptoms and all-cause mortality were identified at baseline. These variables included age, gender (male vs. female), marital status (categorized as married, divorced/separated, widowed, or never married/other), educational level (≥12 years vs. <12 years) and alcohol use (yes vs. no). Health-related variables included self-reported physician-diagnosed major physical diseases recorded in the HRS, such as diabetes, cancer, heart disease and stroke (absence vs. presence).

Psychiatric problems, including depression, insomnia and cognitive impairment, were also measured. Depression was evaluated using the 8-item Center for Epidemiologic Studies Depression Scale (CESD-8) (Van de Velde *et al.*, 2009). The CESD-8 items are (1) feeling depressed, (2) everything was an effort, (3) sleep disturbance, (4) happiness, (5) loneliness, (6) enjoying life, (7) feeling sad and (8) lack of motivation. Each item was scored as 1 or 0 (yes or no), with items 4 and 6 reverse-scored. The total scores ranged from 0 to 8, with higher scores indicating more severe depressive symptoms. A cut-off score of 4 was used to categorize individuals as depressed (Mojtabai and Olfson, 2004).

Insomnia symptoms were measured using the 4-item Jenkins Sleep Scale (JSS-4) (Jenkins *et al.*, 1988), which assessed (1) difficulty initiating sleep, (2) difficulty maintaining sleep, (3) early morning awakenings and (4) feeling rested in the morning. Each item was rated on a 3-point Likert scale ranging from 1 (‘most of the time’) to 3 (‘rarely or never’). Item 4 was reverse-scored. Higher total scores reflected more severe insomnia symptoms, with a cut-off score of 5 used to identify the presence of insomnia (Von Känel *et al.*, 2022).

Cognitive function was assessed using the modified version of the Telephone Interview for Cognitive Status (TICS-m) (Brandt *et al.*, 1988). The TICS-m assesses five cognitive domains: (1) memory (both immediate and delayed recall), (2) attention, (3) calculation, (4) orientation and (5) language. Due to the high rate of missing data in the orientation and language domains, this study focused instead on memory, attention and calculation, following previous research (Crimmins *et al.*, 2011). Memory was assessed through immediate and delayed recall tasks (each scored out of 10), with a total memory score ranging from 0 to 20. Attention was measured using a backward counting task, scored from 0 to 2. Calculation was assessed with a serial subtraction test, with scores ranging from 0 to 5. The total TICS-m score was calculated by summing the scores of these three domains, ranging from 0 to 27, with higher scores indicating better cognitive performance.

### 2.3 Statistical analyses

All statistical analyses were conducted using R (version 4.4.2) (R Core Team, 2020). Frequencies and percentages as well as means and standard deviations (SDs) were used for describing categorical and continuous variables, respectively. All covariates were compared across four loneliness levels using chi-squared and Wilcoxon rank sum tests, as appropriate. Statistical significance was set at an alpha of 0.05 (two-tailed).

#### 2.3.1 Time-to-event analyses

Each participant’s person-time was calculated from the date of their baseline assessment (2010 wave) to either the dates of death ≤85 years or censorship. All-cause mortality rates before the age of 85 years were reported per 1,000 person-years and stratified by loneliness quartiles. To evaluate the association between loneliness severity at baseline and subsequent all-cause mortality before the age of 85 years, Cox proportional hazards regression models were used to calculate hazard ratios (HRs) and 95% confidence intervals (CIs). Three models were constructed, adding covariates progressively. Model 1 adjusted for age and sex; Model 2 added marital status, educational level and alcohol use to Model 1 covariates; and Model 3 further adjusted for history of major physical diseases (i.e. diabetes, cancer, heart conditions and stroke), insomnia (i.e. dichotomized by JSS-4 scores ≥5 or <5), depression (i.e. dichotomized by CESD-8 scores ≥4 or <4) and cognitive function (total TICS-m scores). Loneliness severity, the main exposure of interest, was entered as a categorical variable with the lowest quartile as the reference group.

To assess if the relationship between loneliness and pre-age-85 mortality was moderated by age, we created age-stratified Cox proportional hazards regression models. The groups were 50–64, 65–74 and 75–84 years old. Kaplan–Meier survival curves were plotted to examine potential differences in survival rates across four levels of loneliness and across three different age groups.

Following a previous study (Zhao *et al.*, 2024), we performed time-varying Cox regression models using age as the time scale (Korn *et al.*, 1997), which enable a more precise adjustment for age-specific mortality risks and provide a dynamic perspective on the role of loneliness in mortality before the age of 85 years among middle-aged and older populations. Furthermore, a restricted cubic spline regression with three knots was applied to examine potential nonlinear relationships between loneliness symptoms and risk of mortality before the age of 85 years. To further explore the relationship between individual UCLA-11 items and mortality before the age of 85 years, each item was transformed into a binary variable, with responses coded as 1 (often or some of the time) and 0 (hardly ever or never). All 11 items were entered into Model 3 simultaneously for analysis.

#### 2.3.2 Sensitivity analyses

To assess the robustness of the association between loneliness and mortality before the age of 85 years, four sensitivity analyses were conducted: (1) excluding older individuals at baseline, defined as those within 2 or 5 years of age 85; (2) treating depressive and insomnia symptoms as continuous variables rather than binary variables in Model 3, using revised total CESD-8 scores (excluding item CESD5, ‘Loneliness’, due to its conceptual overlap with the UCLA-11) and the total JSS-4 score, respectively; (3) using the UCLA-3 scale as a binary variable instead of the UCLA-11; and (4) using the complete cases analysis (7,030 participants) without LOCF or NOCF imputations. The UCLA-3 scale is derived from the 20-item UCLA Loneliness Scale and has demonstrated satisfactory reliability and validity consistent with the original scale (Hughes *et al.*, 2004). It assesses participants’ perceptions of (1) lacking companionship, (2) feeling left out and (3) feeling isolated from others. Each item was reverse-scored, with total scores ranging from 3 to 9. Higher scores indicated greater loneliness severity, and a score of 6 or higher was classified as having loneliness symptoms (Qi *et al.*, 2023).

## Results

### 3.1 Participant characteristics

Of the 22,034 HRS participants in the 2010 wave, we excluded 14,248 participants with incomplete or missing UCLA-11 responses (e.g. partial answers or failure to complete the scale), 273 younger than 50 years, 415 age 85 years or older, 2 with data collected posthumously and 704 with missing sociodemographic or health-related information that made imputation unfeasible. The analytical sample consisted of 6,392 participants.

Baseline sociodemographic and health-related characteristics of participants stratified by the four loneliness levels are summarized in Table 1. The mean age of participants was 65.59 years (SD = 9.40), and the majority were female (*n* = 3,630; 56.8%), were married (*n* = 4,131; 64.6%) and had at least a high school education (*n* = 5,487; 85.8%). Univariate analyses indicated significant differences in loneliness levels across most sociodemographic and health-related characteristics (*P* < 0.001), except for a history of cancer (*P* = 0.34).

**Table 1.** Baseline characteristics of participants by severity of loneliness (*N* = 6,392)

Variables	Total (*N* = 6,392)		Loneliness								Univariate analyses	
			Low/no loneliness (Score = 11−13, *N* = 2,167)		Mild loneliness (Score = 14−16, *N* = 1,409)		Moderate loneliness (Score = 17−20, *N* = 1,415)		Severe loneliness (Score = 21−33, *N* = 1,401)			
	*N*	%	*N*	%	*N*	%	*N*	%	*N*	%	*χ*^2^	*P*
Male	2,762	43.2	849	39.2	619	43.9	662	46.8	632	45.1	24.08	**<0.001**
Marital status												
Married	4,131	64.6	1,579	72.9	905	64.2	899	63.5	748	53.4	165.29	**<0.001**
Divorced/separated	1,025	16.0	277	12.8	225	16.0	215	15.2	308	22.0		
Widowed	885	13.8	230	10.6	218	15.5	215	15.2	222	15.8		
Never married/other	351	5.5	81	3.7	61	4.3	86	6.1	123	8.8		
High school education or above (≥12 years of study)	5,487	85.8	1,942	89.6	1,195	84.8	1,195	84.5	1,155	82.4	42.22	**<0.001**
Alcohol use	3,833	60.0	1,368	63.1	843	59.8	826	58.4	796	56.8	16.32	**<0.001**
History of major physical diseases												
Diabetes	1,398	21.9	381	17.6	317	22.5	334	23.6	366	26.1	40.97	**<0.001**
Cancer	939	14.7	316	14.6	216	15.3	220	15.5	187	13.3	3.33	0.34
Heart conditions	1,427	22.3	410	18.9	319	22.6	349	24.7	349	24.9	24.44	**<0.001**
Stroke	324	5.1	77	3.6	68	4.8	81	5.7	98	7.0	22.58	**<0.001**
Depressive symptoms (CESD-–8 ≥ 4)	816	12.8	87	4.0	122	8.7	193	13.6	414	29.6	525.75	**<0.001**
Insomnia symptoms (JSS–-4 ≥ 5)	5,191	81.2	1,600	73.8	1,160	82.3	1,210	85.5	1,221	87.2	127.99	**<0.001**

	Mean	SD	Mean	SD	Mean	SD	Mean	SD	Mean	SD	*Z*	***P***
Age (years)	65.59	9.40	65.78	9.13	66.44	9.55	66.34	9.71	63.66	9.05	79.09*	**<0.001**
Cognitive function (Total TICS-m score)	15.29	4.09	16.02	3.98	15.37	4.12	14.78	4.09	14.59	4.05	135.34*	**<0.001**

*Notes:* Bolded values indicate *P* < 0.05. SD: standard deviation; CESD-8: 8-item Center for Epidemiological Studies Depression Scale; JSS-4: 4-item Jenkins Sleep Scale; TICS-m: modified version of the Telephone Interview for Cognitive Status Scale.

* Analysed using the Wilcoxon rank sum test.

### 3.2 The association between loneliness and mortality before age 85 *years*

#### 3.2.1 Mortality rates before age 85 years

During an average follow-up duration of 7.6 years, a total of 922 deaths were recorded among the 6,392 participants by the 2020 wave. The overall mortality rate before the age of 85 years was 19.1 per 1,000 person-years among midlife and older U.S. adults. Additionally, the mortality rate before the age of 85 years accelerated with age: 9.8 per 1,000 person-years for participants aged 50–64 years, 25.1 per 1,000 person-years for those aged 65–74 years and 42.5 per 1,000 person-years for those aged 75–84 years (Table 3).

#### 3.2.2 The association between loneliness and mortality before oldest old age

**Figure 1.**
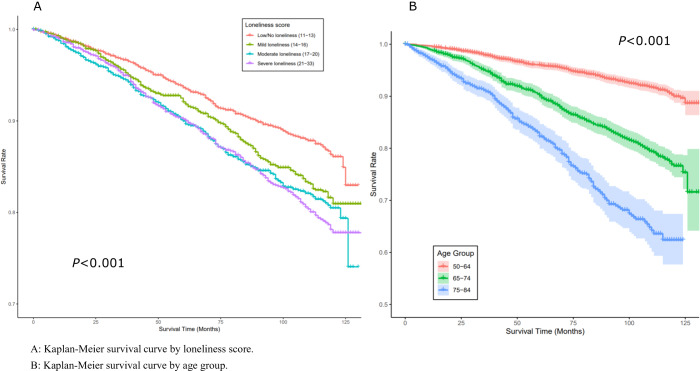
Kaplan–Meier survival curve: (a) Kaplan–Meier survival curve by loneliness score and (b) Kaplan–Meier survival curve by age group.

Column 3 of Table 2 shows a dose–response relationship between loneliness levels and mortality rate before the age of 85 years, and this relationship persists across all three models. In the fully adjusted model, participants with moderate loneliness (UCLA-11 scores 17–20) and severe loneliness (21–33) had 23% and 36% higher risks of all-cause mortality before the age of 85 years, respectively, compared to those with low/no loneliness (11–13) at baseline (adjusted Hazard ratio [aHR] = 1.23, 95% CI = 1.02–1.48 vs. aHR = 1.36, 95% CI = 1.13–1.65). The Kaplan–Meier survival curve (Fig. 1a) illustrates significant differences in survival rates across different loneliness levels (*P* < 0.001).

**Table 2.** The association between loneliness and mortality before the age of 85 years (*N* = 6,392)

Variables	Cases, *N*	Mortality rate, per 1,000 person-years	Model 1			Model 2			Model 3		
			HR	95% CI	*P*	HR	95% CI	*P*	HR	95% CI	*P*
Low/no loneliness (11–13)	238	14.3	Reference			Reference			Reference		
Mild loneliness (14–16)	206	19.6	1.35	1.12–1.63	**0.002**	1.29	1.07–1.56	**0.007**	1.19	0.98–1.43	0.07
Moderate loneliness (17–20)	224	21.6	1.51	1.25–1.81	**<0.001**	1.43	1.19–1.72	**<0.001**	1.23	1.02–1.48	**0.03**
Severe loneliness (21–33)	254	23.5	1.95	1.63–2.33	**<0.001**	1.77	1.48–2.12	**<0.001**	1.36	1.13–1.65	**0.001**
Age (years)			1.09	1.08–1.10	**<0.001**	1.09	1.08–1.10	**<0.001**	1.07	1.06–1.08	**<0.001**
Male			1.40	1.23–1.59	**<0.001**	1.54	1.34–1.76	**<0.001**	1.37	1.19–1.57	**<0.001**
High school education or above (≥12 years)						0.74	0.63–0.87	**<0.001**	0.91	0.77–1.08	0.29
Marital status											
Married						Reference			Reference		
Divorced/separated						1.38	1.15–1.65	**<0.001**	1.34	1.11–1.60	**0.002**
Widowed						1.15	0.95–1.39	0.14	1.11	0.92–1.34	0.27
Never married/other						1.33	0.99–1.78	**0.06**	1.24	0.92–1.66	0.16
Alcohol use						0.63	0.55–0.72	**<0.001**	0.74	0.65–0.85	**<0.001**
History of major physical diseases											
Diabetes									1.48	1.29–1.70	**<0.001**
Cancer									1.80	1.55–2.10	**<0.001**
Heart conditions									1.70	1.48–1.96	**<0.001**
Stroke									1.56	1.27–1.92	**<0.001**
Depressive symptoms (CESD-8 ≥ 4)									1.29	1.08–1.55	**0.006**
Insomnia symptoms (JSS-4 ≥ 5)									1.08	0.90–1.30	0.40
Cognitive function (Total TICS-m score)									0.94	0.93–0.96	**<0.001**

*Notes:* Bolded values: *P* < 0.05

HR: hazard ratio; CI: confidence interval.

Model 1 was adjusted for age and sex.

Model 2 was adjusted for age, sex, marital status, educational level and alcohol consumption.

Model 3 was adjusted for the same covariates as Model 2 plus history of major physical diseases (diabetes, cancer, heart conditions and stroke), insomnia symptoms, depressive symptoms and cognitive function (total TICS-m scores).

The variance inflation factor (VIF) values for the loneliness item of the CESD-8 scale with each item of the UCLA-11 scale were 1.293–2.695, which suggests an absence of multicollinearity. In general, VIF values greater than 10 indicate problematic multicollinearity, while values between 1 and 5 typically reflect low-to-moderate correlation among the variables. Therefore, we used the total CESD-8 score with the cut-off value of 4 indicating participants with depressive symptoms.

In Table 3, this association is stratified by three age groups: 50–64 years, 65–74 years and 75–84 years. Using participants with low or no loneliness (11–13) as the reference group, no significant associations were observed after adjusting for all confounders in the 50–64 age group (Model 3). For the age group of 65–74 years, mild and moderate loneliness was associated with increased mortality in Models 1 and 2, but only severe loneliness demonstrated a consistent and significant association across all models (aHR = 1.37, 95% CI = 1.03–1.83 in Model 3). In contrast, in the 75–84 age group, all levels of loneliness significantly increased mortality risk, with severe loneliness having the highest risk (aHR = 1.77, 95% CI = 1.23–2.56 in Model 3). As expected, Fig. 1b shows that older age groups had lower survival rates compared with younger age groups (*P* < 0.001).

**Table 3.** Associations between loneliness and mortality before the age of 85 years by different age groups (*N* = 6,392)

		Mortality rate, per 1,000 person-years	Model 1			Model 2			Model 3		
	Cases, *N*		HR	95% CI	*P*	HR	95% CI	*P*	HR	95% CI	*P*
Age group (50–64 years) (*N* = 3,119)	258	9.8									
Low/no loneliness (11–13)	64	7.6	Reference			Reference			Reference		
Mild loneliness (14–16)	41	7.6	0.99	0.67–1.46	0.96	0.94	0.63–1.39	0.76	0.84	0.57–1.26	0.40
Moderate loneliness (17–20)	62	11.0	1.43	1.00–2.02	**0.047**	1.31	0.93–1.87	0.13	1.08	0.76–1.55	0.67
Severe loneliness (21–33)	91	13.2	1.74	1.27–2.40	**<0.001**	1.49	1.07–2.06	**0.02**	1.04	0.73–1.48	0.84
Age group (65–74 years) (*N* = 1,951)	390	25.1									
Low/no loneliness (11–13)	111	18.1	Reference			Reference			Reference		
Mild loneliness (14–16)	90	26.1	1.44	1.09–1.90	**0.01**	1.36	1.03–1.80	**0.03**	1.25	0.94–1.66	0.12
Moderate loneliness (17–20)	87	28.3	1.53	1.15–2.03	**0.003**	1.44	1.08–1.91	**0.01**	1.16	0.87–1.56	0.30
Severe loneliness (21–33)	102	35.1	1.96	1.50–2.57	**<0.001**	1.82	1.38–2.39	**<0.001**	1.37	1.03–1.83	**0.03**
Age group (75–84 years) (*N* = 1,322)	274	42.5									
Low/no loneliness (11–13)	63	29.1	Reference			Reference			Reference		
Mild loneliness (14–16)	75	45.5	1.56	1.12–2.18	**0.01**	1.53	1.09–2.14	**0.01**	1.42	1.01–2.00	**0.04**
Moderate loneliness (17–20)	75	45.8	1.55	1.11–2.16	**0.01**	1.55	1.11–2.17	**0.01**	1.47	1.05–2.07	**0.03**
Severe loneliness (21–33)	61	61.5	2.13	1.50–3.03	**<0.001**	2.00	1.40–2.86	**<0.001**	1.77	1.23–2.56	**0.002**

*Notes:* Bolded values: *P* < 0.05; HR: hazard ratio; CI: confidence interval.

Model 1 was adjusted for age and sex.

Model 2 was adjusted for age, sex, marital status, educational level and alcohol consumption.

Model 3 was adjusted for the same covariates as Model 2 plus history of major physical diseases (diabetes, cancer, heart conditions and stroke), insomnia symptoms, depressive symptoms and cognitive function (total TICS-m scores).

**Figure 2.**
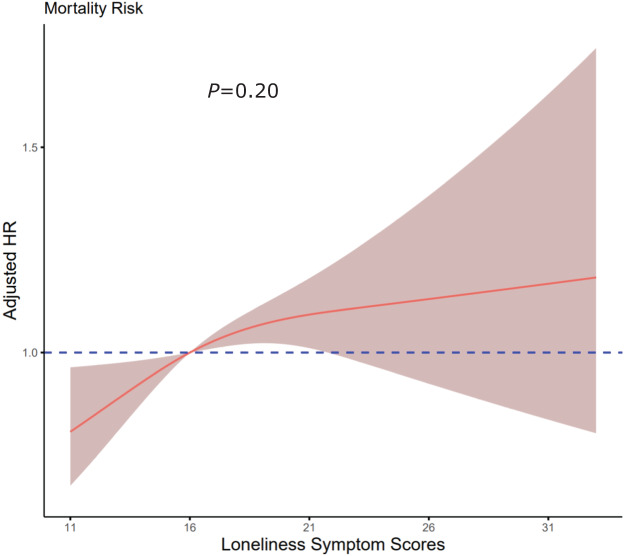
Adjusted hazard ratios of mortality rates according to loneliness symptom scores.

Restricted cubic spline regression analyses with three knots showed a linear and positive association between total loneliness scores and mortality before the age of 85 years (non-linear *P* = 0.20, Fig. 2). Supplementary Table S1 shows the results of time-varying Cox proportion hazards models. In Model 3, a stronger association between severe loneliness and mortality was observed (aHR = 1.65, 95% CI = 1.37–1.99) compared to the unstratified Cox model (aHR = 1.36, 95% CI = 1.13–1.65). Supplementary Table S2 shows that none of the specific loneliness symptoms were significantly associated with mortality before the age of 85 years (all *P* > 0.05).

Graphs show hazard ratios (HRs) according to loneliness symptom scores, adjusted for age, sex, marital status, educational level, living with others, alcohol consumption, insomnia symptoms, depressive symptoms, total TICS-m scores, diabetes, cancer, heart conditions and stroke. Data were analysed using a three-knotted restricted cubic spline Cox proportional hazards regression model. The total UCLA-11 score ranges from 11 to 33, with higher scores indicating more severe loneliness symptoms. Solid lines represent HRs, and shaded areas indicate 95% confidence intervals.

### 3.3 Sensitivity analyses

Consistent associations between loneliness quartiles and mortality before the age of 85 years were observed in Cox proportional hazards regression models: (1) after excluding participants reaching 85 years within 2 and 5 years at baseline (Supplementary Table S3); (2) using revised total CESD-8 scores (excluding item CESD5, ‘Loneliness’) and the total JSS-4 score (Supplementary Table S4); and (3) utilizing the full dataset of 7,030 participants without applying any imputations (Supplementary Table S5). However, when the UCLA-3 scale was used in place of UCLA-11, the association between loneliness and mortality before the age of 85 years in Model 3 was no longer significant (aHR = 1.14; 95% CI = 0.98–1.32; Supplementary Table S6).

## 4. Discussion

To the best of our knowledge, this was the first longitudinal study that explored the association between loneliness and mortality before the age of 85 years among mid- to later-life adults in the United States using a large, nationally representative dataset. The findings revealed a significant dose–response relationship between loneliness and pre-age-85 mortality. After adjusting for a wide range of sociodemographic, lifestyle, physical and mental health factors, moderate and severe loneliness was associated with 23% and 36% higher mortality risks, respectively. Sensitivity analyses confirmed the robustness of these results across various sampling conditions, except when a shorter loneliness instrument was used.

This finding is consistent with previous research showing a significant association between loneliness and an increased risk of all-cause mortality in mid- to later-life populations (Holt-Lunstad *et al.*, 2015; Rico-Uribe *et al.*, 2018). A prior HRS cohort study based on the 2002–2008 waves with 1,604 participants aged 60 years and older similarly found that loneliness was strongly linked to higher mortality risk (Perissinotto *et al.*, 2012). However, our finding contrasts with some studies that found no association between loneliness and mortality (Henriksen *et al.*, 2019; Steptoe *et al.*, 2013; Stessman *et al.*, 2013; Wang *et al.*, 2020). A longitudinal study in the United Kingdom with 2,166 individuals aged 75 years and older found no significant association after adjusting for demographic factors, health conditions and depression (Wang *et al.*, 2020). These discrepancies may arise from differences in covariate adjustment, loneliness assessment and length of follow-up. The present study used two forms of a standard loneliness scale, had a large sample size and had a decade-long follow-up period, which lend credence to our findings.

Our focus on deaths occurring before the age of 85 years may rule out more cases of mortality from purely physical causes, since frailty tends to increase with age. Loneliness likely contributes to early old age mortality through a combination of physiological, behavioural, mental health and social mechanisms. Physiologically, loneliness can induce increased inflammation and elevated stress hormone levels (e.g. cortisol) (Cacioppo *et al.*, 2002), which may impair immune function and raise the risk of chronic diseases like cardiovascular disease and metabolic disorders, ultimately increasing the risk of early mortality (Paul *et al.*, 2021). Behaviourally, individuals experiencing significant loneliness may be more prone to adopting harmful coping strategies such as poor diet, physical inactivity and substance abuse, all of which could have direct negative effects on physical health and increase the risk of early mortality (Hawkley and Cacioppo, 2010). Psychosocially, loneliness may worsen mental health conditions such as depression and anxiety, which are well-established risk factors for mortality (Hawkley and Cacioppo, 2010; Mann *et al.*, 2022) including suicide (Motillon-Toudic *et al.*, 2022). Loneliness reduces social interaction and limits engagement in preventive health behaviours or care-seeking, thereby worsening chronic conditions and increasing vulnerability to acute health events.

An important finding of this study was the variation in the loneliness–mortality relationship across different age groups. After adjusting for health-related factors, no significant association was found between loneliness and early mortality among middle-aged adults (aged 50–64 years). This indicates that loneliness might not be an independent risk factor for mortality in middle-aged adults when other health-related factors (e.g. chronic diseases, depression and cognitive decline) are accounted for. The relatively better health status, stronger social networks, employment-related social interactions, and family responsibilities and support may mitigate the effect of loneliness on mortality, thus nullifying the association. In contrast, severe loneliness predicted early mortality in young-old (65–74 years) adults, as did all loneliness levels (mild, moderate and severe) in old-old (75–84) adults. The time-varying Cox regression model confirms these findings by revealing a stronger relationship between severe loneliness and mortality before the age of 85 years, indicating that the impact of loneliness on mortality increases as individuals age.

Several factors may explain these findings. Although young-old adults may still have relatively stronger social networks and fewer age-related health conditions compared to old-old adults, severe loneliness may still exacerbate underlying health vulnerabilities (e.g. chronic diseases and depression), thereby increasing early mortality risk. In contrast, older-old adults may be facing more health challenges, so a lower level of loneliness can lead to early mortality (World Health Organization, 2023). These health challenges include increased physical comorbidities, cognitive decline, depressive symptoms as well as diminished social networks and support due to retirement or the loss of loved ones (Cudjoe *et al.*, 2020; National Academies of Sciences *et al.*, 2020), which makes them more vulnerable to the negative consequences of loneliness and to a higher mortality risk even at lower levels of loneliness (Luo *et al.*, 2012).

Our findings have important clinical implications. First, a routine screening for loneliness should be incorporated into healthcare assessments for all mid- to later-life adults, particularly for those undergoing major life transitions. Second, for young-old adults experiencing severe loneliness, early interventions are necessary to prevent or reduce the risk of negative health effects. Unlike old-old adults who are expected to be retired, middle-aged adults are generally expected to have jobs. For example, the loss of employment (or lack of job opportunities) may lead to prolonged social withdrawal, a phenomenon called ‘hikikomori’ that was first observed in Japan but also present in other countries (Kato *et al.*, 2019, 2012).

For old-old adults, a more comprehensive care plan is required to actively address all levels of loneliness, as it is a key risk factor for early mortality (National Academies of Sciences *et al.*, 2020). Enhancing social skills, improving social support, increasing social opportunities and altering maladaptive social cognition are recommended as these multi-faceted approaches are found to be more effective than those focusing on a single aspect (Patil and Braun, 2024). Specifically, certain interventions, such as animal therapy, psychological therapy and skill-building activities, have proven to be more effective than strategies centred solely on social facilitation or health promotion (Hoang *et al.*, 2022; Patil and Braun, 2024). These targeted and age-specific approaches are essential to reduce the mortality risks associated with loneliness, ultimately improving quality of life and health outcomes for older adults.

Although the UCLA-11 total score showed a significant association with mortality risk, individual loneliness symptoms were not linked to mortality before the age of 85 years. This finding indicates that the global effect of multiple loneliness symptoms, rather than any individual symptom alone, may be more predictive of early mortality risk. Sensitivity analysis using the UCLA-3 scale as a dichotomous variable revealed no significant associations. This discrepancy aligns with a study (Raymo and Wang, 2022) that found different associations of loneliness measured by the UCLA-11 and UCLA-3 with sociodemographic factors and health outcomes. Differences in scale length and content may account for these variations. While the UCLA-3 scale is practical for quick assessments, it may not fully capture the cluster of symptoms, focusing solely on social isolation (Hughes *et al.*, 2004). In contrast, the UCLA-11 scale incorporates additional dimensions, such as available social connections and a sense of belonging (Lee and Cagle, 2017), and is thus a more comprehensive assessment and more sensitive to subtle associations of loneliness with mortality. These findings indicated that loneliness measurement tools may influence results, highlighting the need for standardized assessments to be used in future research. Future research should consider the choice of suitable measurement scales when examining the impact of loneliness on health outcomes, balancing the need for precision with practicality.

This study has several strengths including the use of a large, representative dataset and robust statistical models that controlled for a wide range of confounders. The longitudinal design enables temporal inference, providing stronger evidence for a potential causal relationship between loneliness and mortality before the age of 85 years. However, several limitations should be noted. First, certain confounding variables related to both loneliness and mortality, such as social isolation, social support, early-life social experiences and functional limitations, were not included in the analysis due to numerous missing data in the HRS. Second, relying on self-reported data in this study may have introduced potential recall or social desirability biases. Third, although previous research demonstrated that loneliness can be classified into transient and chronic subtypes, with each having distinct impacts on health outcomes (e.g. cognitive decline) (Zhong *et al.*, 2016), our study measured loneliness only at baseline, which may limit the ability to capture its dynamic nature and may potentially underestimate the cumulative or changing effects of loneliness on early mortality. Future studies with repeated assessments of loneliness are needed to better differentiate the health impacts of chronic versus transient loneliness and to clarify their mechanisms in relation to early mortality risk. Fourth, the study population was drawn from the United States, which may limit the generalizability of findings to other cultural contexts. Future research could explore whether these associations existed in diverse populations and consider how cultural factors might shape the interplay between loneliness and health outcomes. Fifth, we examined the interaction between the severity of loneliness and gender and found no significant effect modification, perhaps partly due to limited statistical power. Future research should examine more detailed interaction analyses in larger and more diverse samples or should include more comprehensive covariate data (e.g. socioeconomic status and race/ethnicity) to better understand potential heterogeneity in the association between loneliness and early mortality. Finally, using the age of 85 years as a binary cut-off might introduce artificial censoring and may misclassify mortality outcomes among the oldest old. Although we conducted sensitivity analyses excluding individuals close to the age of 85 years at baseline to mitigate this issue, this approach, which is widely used in both clinical practice and research, may still underestimate mortality risk in very old age and limit the ability to capture mortality dynamics beyond this age.

In conclusion, this study highlights loneliness as a significant predictor of mortality before the age of 85 years, particularly among older adults. By focusing on this critical age threshold, the findings contribute to a deeper understanding of the health impacts of loneliness and emphasize the urgent need to address this public health issue. Future research could explore targeted interventions and underlying mechanisms to mitigate the effects of loneliness and enhance health longevity and quality of life among mid- to later-life populations.

## Supporting information

10.1017/S2045796025100188.sm001 Fan et al. supplementary material Fan et al. supplementary material

## Data Availability

The data that support the findings of this study are available from the University of Michigan Health and Retirement Study Platform at https://hrsdata.isr.umich.edu/data-products/public-survey-data.

## Supplementary material

The supplementary material for this article can be found at https://doi.org/10.1017/S2045796025100188.
