# Supplementary material for: Predictive role of loneliness on mortality before the age 85 years among mid- to later-life adults in the United States: a 10-year retrospective cohort study
Source: Epidemiol Psychiatr Sci. 2025 Sep 11;34:e48. doi: 10.1017/S2045796025100188 (PMC12450537; doi:10.1017/S2045796025100188)
Supplement: Fan et al. supplementary material [file S2045796025100188sup001.docx]

**Supplementary materials**

Table S1. Association of loneliness and mortality before age 85 years using time-varying Cox proportion hazards models (N=6,392)

Table S2. The associations between specific loneliness symptoms and mortality before age 85 years (N=6,392)

Table S3. Associations between loneliness symptoms and mortality before age 85 years excluding people within 2 and 5 years to age 85.

Table S4. Associations between loneliness symptoms and mortality before age 85 years with revised covariates of total JSS-4 score and CESD-8 score excluding the item CESD5 (loneliness) (N=6,392)

Table S5. Associations between loneliness symptoms and mortality before age 85 years using UCLA-3 scale (N=6,392)

Table S6. Associations between loneliness symptoms and mortality before age 85 years in complete data without imputations (N=7,030)

**Table S1. The association of loneliness and mortality before age 85 years using time-varying Cox proportion hazards models^a^ (N=6,392)**

|  | **Model 1** | | | **Model 2** | | | **Model 3** | | |
| --- | --- | --- | --- | --- | --- | --- | --- | --- | --- |
|  | **HR** | **95%CI** | ***P*** | **HR** | **95%CI** | ***P*** | **HR** | **95%CI** | ***P*** |
| Low/No loneliness (11-13) | Reference | | | Reference | | | Reference | | |
| Mild loneliness (14-16) | 1.15 | 0.96-1.39 | 0.14 | 1.13 | 0.94-1.37 | 0.19 | 1.05 | 0.87-1.27 | 0.59 |
| Moderate loneliness (17-20) | 1.22 | 1.02-1.47 | **0.03** | 1.24 | 1.03-1.49 | **0.02** | 1.13 | 0.94-1.36 | 0.21 |
| Severe loneliness (21-33) | 2.06 | 1.73-2.46 | **<0.001** | 1.93 | 1.62-2.32 | **<0.001** | 1.65 | 1.37-1.99 | **<0.001** |
| Male | 1.39 | 1.20-1.56 | **<0.001** | 1.35 | 1.171.54 | **<0.001** | 1.28 | 1.11-1.47 | **<0.001** |
| High school education or above (≥12 years) |  |  |  | 0.87 | 0.74-1.03 | 0.10 | 0.92 | 0.78-1.09 | 0.34 |
| Marital status |  |  |  |  |  |  |  |  |  |
| Married |  |  |  | Reference | | | Reference | | |
| Divorced/Separated |  |  |  | 2.02 | 1.68-2.42 | **<0.001** | 2.03 | 1.69-2.43 | **<0.001** |
| Widowed |  |  |  | 0.72 | 0.60-0.87 | **<0.001** | 0.71 | 0.59-0.86 | **<0.001** |
| Never married/Other |  |  |  | 1.95 | 1.45-2.62 | **<0.001** | 1.93 | 1.43-2.59 | **<0.001** |
| Alcohol use |  |  |  | 0.78 | 0.69-0.89 | **<0.001** | 0.86 | 0.75-0.98 | **0.03** |
| History of major physical diseases |  |  |  |  |  |  |  |  |  |
| Diabetes |  |  |  |  |  |  | 1.54 | 1.34-1.77 | **<0.001** |
| Cancer |  |  |  |  |  |  | 1.13 | 0.97-1.31 | 0.12 |
| Heart conditions |  |  |  |  |  |  | 1.21 | 1.06-1.39 | **0.006** |
| Stroke |  |  |  |  |  |  | 1.40 | 1.14-1.72 | **0.001** |
| Depressive symptoms (CESD-8 ≥ 4) |  |  |  |  |  |  | 1.44 | 1.20-1.72 | **<0.001** |
| Insomnia symptoms (JSS-4 ≥ 5) |  |  |  |  |  |  | 1.08 | 0.89-1.29 | 0.45 |
| Total TICS-m score |  |  |  |  |  |  | 1.00 | 0.98-1.01 | 0.61 |
| Notes. HR: Hazard ratio; CI: Confidence interval. Bolded values: *P*< 0.05.  ^a^ Time-varying Cox proportion hazards models using age as the time scale.  Model 1 was adjusted for sex.  Model 2 was adjusted for sex, marital status, educational level, and alcohol consumption.  Model 3 was adjusted for the same covariates as model 2 plus History of major physical diseases (diabetes, cancer, heart conditions and stroke), insomnia symptoms, depressive symptoms and total TICS-m scores. | | | | | | | | | |

**Table S2. The associations between specific loneliness symptoms and mortality before age 85 years (N=6,392)**

| **Loneliness symptom items** | **N** | **%** | **HR** | **95%CI** | ***P*** |
| --- | --- | --- | --- | --- | --- |
| Lack companionship | 2,822 | 44.2 | 0.88 | 0.74-1.05 | 0.15 |
| Feel left out | 2,660 | 41.6 | 0.96 | 0.80-1.15 | 0.63 |
| Feel isolated from others | 2,079 | 32.5 | 1.15 | 0.95-1.39 | 0.15 |
| In tune with others | 3,326 | 52.0 | 1.01 | 0.87-1.18 | 0.89 |
| Alone | 2,691 | 42.1 | 1.13 | 0.95-1.34 | 0.17 |
| People can talk to | 2,277 | 35.6 | 1.13 | 0.91-1.39 | 0.27 |
| People can turn to | 2,317 | 36.3 | 0.94 | 0.75-1.17 | 0.55 |
| People understand you | 3,224 | 50.4 | 0.87 | 0.72-1.04 | 0.12 |
| People feel close to | 2,103 | 32.9 | 1.10 | 0.91-1.33 | 0.31 |
| Feel part of group | 3,313 | 51.8 | 1.06 | 0.89-1.27 | 0.51 |
| A lot in common with friends | 3,384 | 52.9 | 1.13 | 0.95-1.35 | 0.17 |
| Notes. HR: Hazard ratio; CI: Confidence Interval. Each item was assessed using a binary variable, with 0 indicating 'hardly ever or never' and 1 indicating 'some of the time or often’. Model was adjusted for the individual loneliness symptoms of UCLA-11, age, sex, marital status, educational level, alcohol consumption, insomnia symptoms, depressive symptoms, total TICS-m scores, and history of major physical diseases (diabetes, cancer, heart conditions and stroke). | | | | | |

**Table S3. The association between loneliness symptoms and mortality before age 85, excluding people within 2 or 5 years of age 85**

|  | **Cases,**  **N** | **Mortality rate, per 1000**  **person-years** | **Model 1** | | | **Model 2** | | | **Model 3** | | |
| --- | --- | --- | --- | --- | --- | --- | --- | --- | --- | --- | --- |
|  |  |  | **HR** | **95%CI** | ***P*** | **HR** | **95%CI** | ***P*** | **HR** | **95%CI** | ***P*** |
| Excluding 2 years to age 85 (N=6,178) | 902 | 18.8 |  |  |  |  |  |  |  |  |  |
| Low/No loneliness  (11-13) | 233 | 14.0 | Reference | | | Reference | | | Reference | | |
| Mild loneliness  (14-16) | 199 | 19.1 | 1.34 | 1.11-1.62 | **0.002** | 1.28 | 1.06-1.55 | **0.01** | 1.17 | 0.97-1.42 | 0.10 |
| Moderate loneliness (17-20) | 219 | 21.3 | 1.52 | 1.26-1.83 | **<0.001** | 1.44 | 1.20-1.73 | **<0.001** | 1.23 | 1.02-1.49 | **0.03** |
| Severe loneliness (21-33) | 251 | 23.3 | 1.96 | 1.64-2.35 | **<0.001** | 1.77 | 1.48-2.13 | **<0.001** | 1.36 | 1.12-1.64 | **0.002** |
| Excluding 5 years to age 85 (N=5,856) | 862 | 18.4 |  |  |  |  |  |  |  |  |  |
| Low/No loneliness  (11-13) | 226 | 13.9 | Reference | | | Reference | | | Reference | | |
| Mild loneliness  (14-16) | 188 | 18.6 | 1.33 | 1.10-1.62 | **0.004** | 1.27 | 1.04-1.54 | **0.02** | 1.17 | 0.96-1.43 | 0.11 |
| Moderate loneliness (17-20) | 205 | 20.5 | 1.49 | 1.23-1.80 | **<0.001** | 1.40 | 1.16-1.70 | **<0.001** | 1.19 | 0.98-1.45 | 0.08 |
| Severe loneliness (21-33) | 243 | 23.0 | 1.97 | 1.64-2.36 | **<0.001** | 1.76 | 1.46-2.12 | **<0.001** | 1.33 | 1.09-1.62 | **0.004** |
| Notes. HR: Hazard ratio; CI: Confidence interval. Bolded values: *P*< 0.05.  Model 1 was adjusted for sex.  Model 2 was adjusted for sex, marital status, educational level, and alcohol consumption.  Model 3 was adjusted for the same covariates as model 2 plus history of major physical diseases (diabetes, cancer, heart conditions and stroke), insomnia symptoms, depressive symptoms and total TICS-m scores. | | | | | | | | | | | |

**Table S4. The association between loneliness symptoms and mortality before age 85 years with revised covariates of total JSS-4 score and CESD-8 score excluding the item CESD5 (loneliness) (N=6,392)**

|  | **HR** | **95%CI** | ***P*** |
| --- | --- | --- | --- |
| Low/No loneliness (11-13) | Reference | | |
| Mild loneliness (14-16) | 1.17 | 0.97-1.41 | 0.10 |
| Moderate loneliness (17-19) | 1.21 | 1.00-1.45 | **0.048** |
| Severe loneliness (20-33) | 1.29 | 1.06-1.56 | **0.01** |
| Notes. HR: Hazard ratio; CI: Confidence interval. Bolded values: *P*< 0.05.  Model was adjusted for age, sex, marital status, educational level, alcohol consumption, history of major physical diseases (diabetes, cancer, heart conditions and stroke), total JSS-4 scores, total CESD-8 score excluding the item CESD5 (loneliness) and total TICS-m scores. | | | |

**Table S5. The association between loneliness symptoms and mortality before age 85 years using UCLA-3 scale (N=6,392)**

|  | **Cases,**  **N** | **Mortality rate, per 1000**  **person-years** | **Model 1** | | | **Model 2** | | | **Model 3** | | |
| --- | --- | --- | --- | --- | --- | --- | --- | --- | --- | --- | --- |
|  |  |  | **HR** | **95%CI** | ***P*** | **HR** | **95%CI** | ***P*** | **HR** | **95%CI** | ***P*** |
| Without loneliness symptoms  (UCLA-3 <6, N=4,780) | 646 | 17.9 | Reference | | | Reference | | | Reference | | |
| With loneliness  Symptoms  (UCLA-3 ≥6, N=1,612) | 276 | 22.3 | 1.50 | 1.30-1.73 | **<0.001** | 1.38 | 1.19-1.60 | **<0.001** | 1.14 | 0.98-1.32 | 0.10 |
| Notes. HR: Hazard ratio; CI: Confidence interval. Bolded values: *P*< 0.05.  Model 1 was adjusted for age and sex.  Model 2 was adjusted for age, sex, marital status, educational level, and alcohol consumption.  Model 3 was adjusted for the same covariates as model 2 plus history of major physical diseases (diabetes, cancer, heart conditions and stroke), insomnia symptoms, depressive symptoms and total TICS-m scores. | | | | | | | | | | | |

**Table S6. The association between loneliness symptoms and mortality before age 85 years in complete data without imputations (N=7,030)**

|  | **Cases,**  **N** | **Mortality rate, per 1000**  **person-years** | **Model 1** | | | **Model 2** | | | **Model 3** | | |
| --- | --- | --- | --- | --- | --- | --- | --- | --- | --- | --- | --- |
|  |  |  | **HR** | **95%CI** | ***P*** | **HR** | **95%CI** | ***P*** | **HR** | **95%CI** | ***P*** |
| Low/No loneliness  (Score=11-13, N=2,338) | 244 | 13.7 | Reference | | | Reference | | | Reference | | |
| Mild loneliness  (Score=14-16, N=1,537) | 212 | 18.8 | 1.34 | 1.11-1.61 | **0.002** | 1.33 | 1.10-1.62 | **0.004** | 1.23 | 0.99-1.52 | 0.06 |
| Moderate loneliness (Score=17-20, N=1,564) | 229 | 20.4 | 1.47 | 1.22-1.76 | **<0.001** | 1.41 | 1.17-1.72 | **<0.001** | 1.24 | 1.00-1.53 | **0.048** |
| Severe loneliness (Score=21-33, N=1,591) | 276 | 23.1 | 1.95 | 1.64-2.31 | **<0.001** | 1.79 | 1.48-2.17 | **<0.001** | 1.32 | 1.06-1.65 | **0.02** |
| Notes. HR: Hazard ratio; CI: Confidence interval. Bolded values: *P*< 0.05.  Model 1 was adjusted for age and sex.  Model 2 was adjusted for age, sex, marital status, educational level, and alcohol consumption. Totally 1,584 samples with missing data in analyzing variables were deleted.  Model 3 was adjusted for the same covariates as model 2 plus history of major physical diseases (diabetes, cancer, heart conditions and stroke), insomnia symptoms, depressive symptoms and total TICS-m scores. Totally 2,634 samples with missing data in analyzing variables were deleted. | | | | | | | | | | | |
